# Quantum medicine: A quantum–mechanical framework for redox biology, disease and precision medicine

**DOI:** 10.1002/ctm2.70598

**Published:** 2026-01-19

**Authors:** Ji‐Yong Sung, Jae‐Ho Cheong

**Affiliations:** ^1^ Department of Neurosurgery Seoul National University Bundang Hospital Seongnam‐si Republic of Korea; ^2^ Department of Surgery Yonsei University College of Medicine Seoul Republic of Korea

**Keywords:** biomedical applications of quantum technologies, precision medicine, quantum biology, quantum biomedicine, quantum computing, redox biology

## Abstract

**Background:**

Key biological processes underlying health and disease‐including electron transfer, redox regulation, and radical‐mediated signaling‐are fundamentally governed by quantum‐mechanical principles. These processes are central to mitochondrial function, metabolism, and cellular signaling, yet their biomedical implications have remained difficult to address using classical computational approaches.

**Rationale:**

Recent advances in quantum computing, quantum sensing, and quantum machine learning enable direct simulation and measurement of quantum phenomena in biologically relevant systems. Hybrid quantum‐classical algorithms, such as the Variational Quantum Eigensolver and Quantum Phase Estimation, now provide first‐principles access to redox potentials, electronic couplings, and spin‐dependent reactions that are directly linked to disease mechanisms. These developments establish the foundation for quantum biomedicine as a translational framework bridging molecular physics and clinical medicine.

**Content:**

This review synthesizes current progress in the application of quantum technologies to biomedicine, emphasizing translational relevance. We discuss quantum‐informed modeling of cancer metabolism and redox rewiring, protein misfolding in neurodegenerative diseases, immune and inflammatory signaling, infectious disease mechanisms, and drug discovery. We further propose a Quantum‐Experimental‐Clinical (QEC) pipeline that integrates quantum simulations with experimental validation and multi‐omics clinical data, enabling mechanistic interpretation of disease phenotypes and identification of redox‐ and spin‐sensitive therapeutic targets.

**Conclusion:**

Quantum biomedicine introduces a new mechanistic layer that links electronic‐scale processes to clinical phenotypes. While current implementations are constrained by NISQ‐era hardware, rapid advances in quantum algorithms and sensing technologies position quantum approaches as emerging tools in precision and translational medicine. Strategic integration of quantum methods with experimental and clinical workflows may accelerate biomarker discovery and therapeutic development.

**Key points:**

- Quantum biomedicine redefines life as a dynamic equilibrium sustained by quantum coherence, tunnelling and redox resonance.
- Hybrid quantum–classical algorithms, such as VQE and QPE, enable first‐principles modelling of redox and spin‐dependent reactions with near‐experimental accuracy.
- NISQ‐era hardware supports proof‐of‐concept simulations of electron tunnelling and radical‐pair dynamics, bridging computation with measurable biophysics.
- Integration of quantum simulations with spectroscopy and cryo‐EM establishes a quantum–experimental–clinical (QEC) pipeline linking theory, experiment and medicine.
- Ethical, educational and governance frameworks are essential for equitable, transparent and sustainable implementation of quantum health technologies.

## Graphical abstract

Quantum medicine integrates quantum simulation, experimental validation and clinical data into a unified QEC pipeline. Quantum algorithms compute electronic and spin‐level properties of redox biology, which are validated by quantum sensing and structural biology and translated into precision diagnostics and therapy through multi‐omics and quantum machine learning.

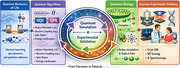

## 1 INTRODUCTION

Biological systems are not merely influenced by quantum mechanics‐they are quantum systems in the most literal sense.^1^ Many processes essential to life, including electron tunnelling in respiratory complexes,^2^ proton‐coupled transfer^3^ in metabolic enzymes, quantum coherence in photosynthesis and spin dynamics in radical signalling,^4^ are inherently quantum mechanical in nature.^5^ Historically, however, these phenomena have been treated as peripheral or irrelevant in biomedical research. This neglect stemmed not from a lack of biological significance, but from the computational limitations of classical tools. Traditional approaches, such as molecular dynamics (MD), empirical force fields and density functional theory (DFT), have been indispensable in advancing biochemistry and molecular medicine. Yet, they rely on approximations that cannot capture essential features such as quantum entanglement, non‐locality and electron correlation‐phenomena that fundamentally govern redox regulation, enzymatic catalysis, signal transduction and metabolic reprogramming.^6^, ^7^

The emergence of quantum computing represents a paradigm shift in how we model biological complexity. Instead of approximating wavefunctions indirectly, quantum computers can encode them directly into qubits, allowing molecular systems to be simulated in their native quantum mechanical language. Quantum‐native algorithms‐including the variational quantum eigensolver (VQE), quantum phase estimation (QPE) and quantum imaginary time evolution (QITE)‐are specifically designed to solve strongly correlated, multireference and excited‐state problems that lie beyond the reach of classical computation. This transformative capability is already beginning to materialize as noisy intermediate‐scale quantum (NISQ) devices have successfully modelled bioinorganic clusters with realistic accuracy, predicting redox potentials within approximately  .1 eV of experimental values.^8^ Such achievements signal the dawn of quantum biomedicine, a new field that integrates quantum computation, quantum sensing and quantum machine learning (QML) to explore the molecular foundations of health and disease.^9^, ^10^, ^11^

**FIGURE 1:**
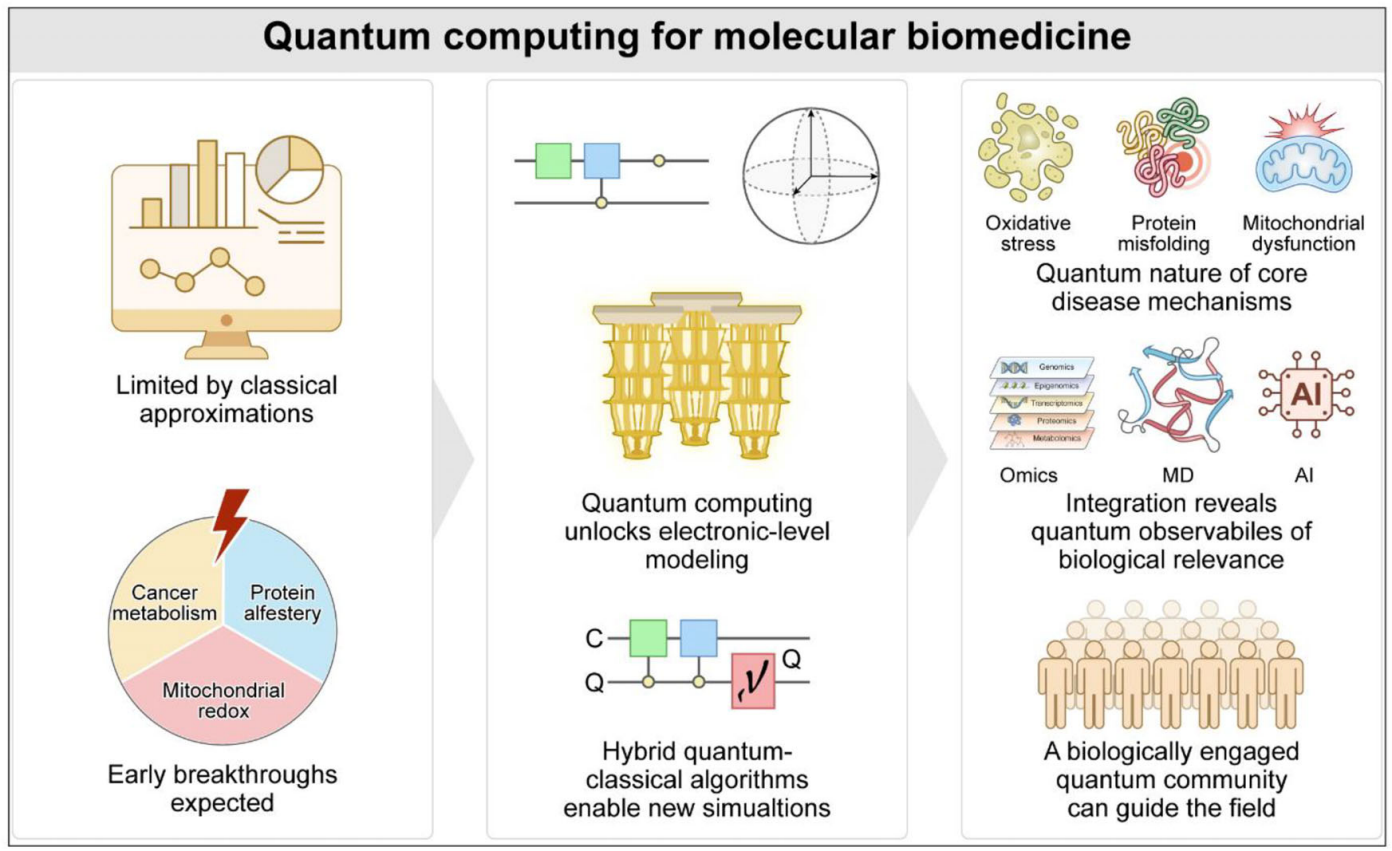
Opportunities for quantum computing in molecular biomedicine. This schematic illustrates how quantum computing can revolutionize the modelling of biological systems by overcoming limitations of classical approximations. Classical simulations often fall short in accurately capturing electronic‐level processes fundamental to biology. Quantum computing enables first‐principles modelling of molecular systems, unlocking insights into the quantum nature of disease mechanisms such as oxidative stress, protein misfolding and mitochondrial dysfunction. Hybrid quantum–classical algorithms offer a path toward new biologically relevant simulations, integrating omics, molecular dynamics (MD) and artificial intelligence (AI) data. Key areas where early breakthroughs are expected include cancer metabolism, protein allostery and mitochondrial redox chemistry. Progress in this field will depend on a biologically engaged quantum computing community to guide and interpret developments.

This review aims to provide a forward‐looking perspective on how quantum technologies are reshaping the biomedical landscape. Figure 1 provides a conceptual overview of this paradigm shift‐illustrating how quantum computing transcends classical limitations to enable electronic‐level modelling of biological systems, thereby illuminating quantum underpinnings of disease mechanisms and guiding early biomedical breakthroughs.

We focus on three domains where early breakthroughs are most likely and most impactful: (i) redox rewiring and metabolic regulation in cancer; (ii) protein misfolding, allostery and conformational transitions in disease‐associated proteins. We further examine how hybrid quantum–classical algorithms bridge omics data, structural biology and molecular simulations to deliver mechanistic insights that were previously inaccessible. Finally, we discuss the emerging role of quantum sensing and QML as complementary tools in biomedical discovery and highlight future challenges and opportunities for interdisciplinary collaboration. Across Chapters 3–5, we show how hybrid quantum–classical algorithms link quantum simulations with experimental and clinical validation‐forming a quantum–experimental–clinical (QEC) pipeline that bridges omics data, structure and function toward a quantum–informed model of disease. Together, these advances outline a coherent roadmap for the next era of molecular medicine‐one grounded in the quantum nature of life itself.^12^, ^13^

## 2 CORE PRINCIPLES AND COMPUTATIONAL FRAMEWORKS OF QUANTUM COMPUTING

Quantum computing departs fundamentally from classical computation by directly harnessing quantum phenomena to process information. Before exploring its biomedical applications, this section establishes the conceptual foundation by outlining the essential principles of quantum information and the computational frameworks that make such approaches possible. We introduce the key quantum properties relevant to molecular modelling, clarify how they differ from classical paradigms and set the stage for understanding how these principles enable powerful new strategies in biomedical research.

### 2.1 Fundamentals of quantum information

Quantum computing is built upon fundamental principles of quantum mechanics‐superposition, entanglement and interference‐which enable computational capabilities far beyond those of classical systems. Unlike classical bits, which represent either 0 or 1, quantum bits (qubits) exist in a superposition of states, allowing them to encode a continuum of information. This property exponentially expands the computational space: An *n*‐qubit system can represent 2*^n^* states simultaneously, enabling parallel exploration of complex solution spaces that would be intractable for classical computers.^14^, ^15^, ^16^

Entanglement,^17^, ^18^ a uniquely quantum correlation between qubits, allows operations on one qubit to instantaneously affect the state of another, regardless of distance. This non‐classical connectivity enables information propagation through entanglement‐mediated correlations, reducing circuit depth and enabling quantum algorithms to explore exponentially large Hilbert spaces more efficiently than classical architectures.

Quantum interference, the constructive and destructive combination of probability amplitudes, is exploited to amplify correct solutions while suppressing incorrect ones during computation.^18^

Together, these principles allow quantum computers to solve certain problems‐such as factoring large integers, simulating quantum systems and optimizing high‐dimensional functions‐with exponential speedups or qualitatively new capabilities compared to classical algorithms.^19^, ^20^, ^21^

### 2.2 Historical development and biomedical relevance

#### 2.2.1 From quantum theory to quantum computing

The foundations of quantum computing are deeply rooted in the early 20th‐century revolution in physics that reshaped our understanding of matter and energy. Quantum mechanics,^22^ formulated between 1900 and 1930 by pioneers such as Max Planck, Albert Einstein, Niels Bohr, Werner Heisenberg and Erwin Schrödinger, revealed that particles at the atomic and subatomic scales do not obey classical deterministic laws. Instead, their behaviour is governed by probability amplitudes, wavefunctions and discrete quantized states. These principles‐superposition, entanglement and quantum coherence‐later became the theoretical pillars upon which quantum information science is built.^23^, ^24^, ^25^

The idea of using quantum mechanics for computation emerged much later. In the 1980s, Richard Feynman famously argued that classical computers could not efficiently simulate quantum systems because the computational cost grows exponentially with system size. He proposed the concept of a ‘quantum simulator’‐a device that could use quantum mechanical principles to model other quantum systems directly.^26^ This was followed by Deutsch, who formalized the concept of a universal quantum computer capable of performing any computation that a classical computer could, but potentially much faster.^27^, ^28^

A major milestone came in the 1990s with the development of quantum algorithms that demonstrated dramatic speedups. Shor^29^ showed that quantum computers could factor large integers exponentially faster than classical methods, whereas Grover^30^ developed a quantum search algorithm offering quadratic speedup. These breakthroughs transformed quantum computing from a theoretical curiosity into a potentially transformative technology.^30^, ^31^, ^32^, ^33^

#### 2.2.2 The rise of quantum simulation and molecular applications

By the early 2000s, researchers recognized that one of the most promising near‐term applications of quantum computing lay in quantum simulation‐the ability to model the behaviour of complex quantum systems such as molecules, materials and biological macromolecules. Early proof‐of‐principle experiments demonstrated that even a handful of qubits could approximate simple molecular Hamiltonians, such as the hydrogen molecule (H_2_), using quantum algorithms like the VQE and QPE.^34^

By encoding molecular wavefunctions directly into qubits, quantum hardware bypasses the exponential scaling barrier that limits classical methods such as DFT or coupled‐cluster calculations. This capability is particularly relevant in biochemistry and molecular biology, where electron correlation, multireference character and non‐local quantum effects often determine function and reactivity.^35^, ^36^, ^37^, ^38^

### 2.3 Quantum algorithms and hybrid approaches

#### 2.3.1 Biomedical relevance: from fundamental physics to life sciences

The intersection of quantum computing and biomedicine arises from a simple yet profound observation: biological systems are quantum systems. Many of the most fundamental processes underlying life‐such as electron transfer (ET) in respiration, proton tunnelling in enzymatic catalysis, spin‐dependent radical reactions and coherent energy transport in photosynthesis—are inherently quantum mechanical. Traditional computational biology methods, while powerful, rely on classical approximations that often fail to capture these phenomena at the level of first principles.^39^, ^40^

Early applications of quantum computing to biology focused primarily on quantum chemistry of biomolecules, such as small peptides, cofactors and metal centres. Modern quantum computing in biomedicine is intrinsically hybrid. Quantum biomedicine is implemented through hybrid quantum–classical pipelines that connect qubit‐level simulation with classical omics, imaging and clinical data. Quantum models of proton‐coupled electron transfer (PCET) in metabolic enzymes have provided mechanistic insights into reaction kinetics and energy barriers that directly relate to cancer metabolism and drug resistance.^41^, ^42^, ^43^, ^44^, ^45^, ^46^, ^47^, ^48^, ^49^ As hardware improved, the biomedical scope expanded. Recent studies have used hybrid quantum–classical algorithms to explore protein folding landscapes, predict mutation‐induced conformational changes and simulate the electronic consequences of cancer‐associated mutations in genes such as p53 and KRAS. Quantum‐enhanced classifiers have also shown promise in analysing high‐dimensional omics datasets, improving cancer subtype classification accuracy and biomarker discovery compared to classical machine learning methods.^50^, ^51^, ^52^, ^53^

Quantum computing inherits the ambitions of quantum chemistry but extends them into living systems.^54^ Classical quantum chemistry, even with high‐performance computing, becomes infeasible for biomolecules beyond a few dozen atoms. The complexity scales exponentially due to electron correlation and orbital interactions.^55^ Quantum algorithms provide a route around this bottleneck by naturally encoding the molecular Hamiltonian into qubit registers. This allows the treatment of biological cofactors, metalloproteins and enzyme active sites with a degree of accuracy previously unattainable.^56^, ^57^, ^58^ To improve accessibility for biomedical researchers, we explicitly link core quantum algorithms to specific biological use cases. The VQE is particularly well suited for simulating the electronic structure of redox‐active cofactors under biologically relevant constraints.^59^

**FIGURE 2:**
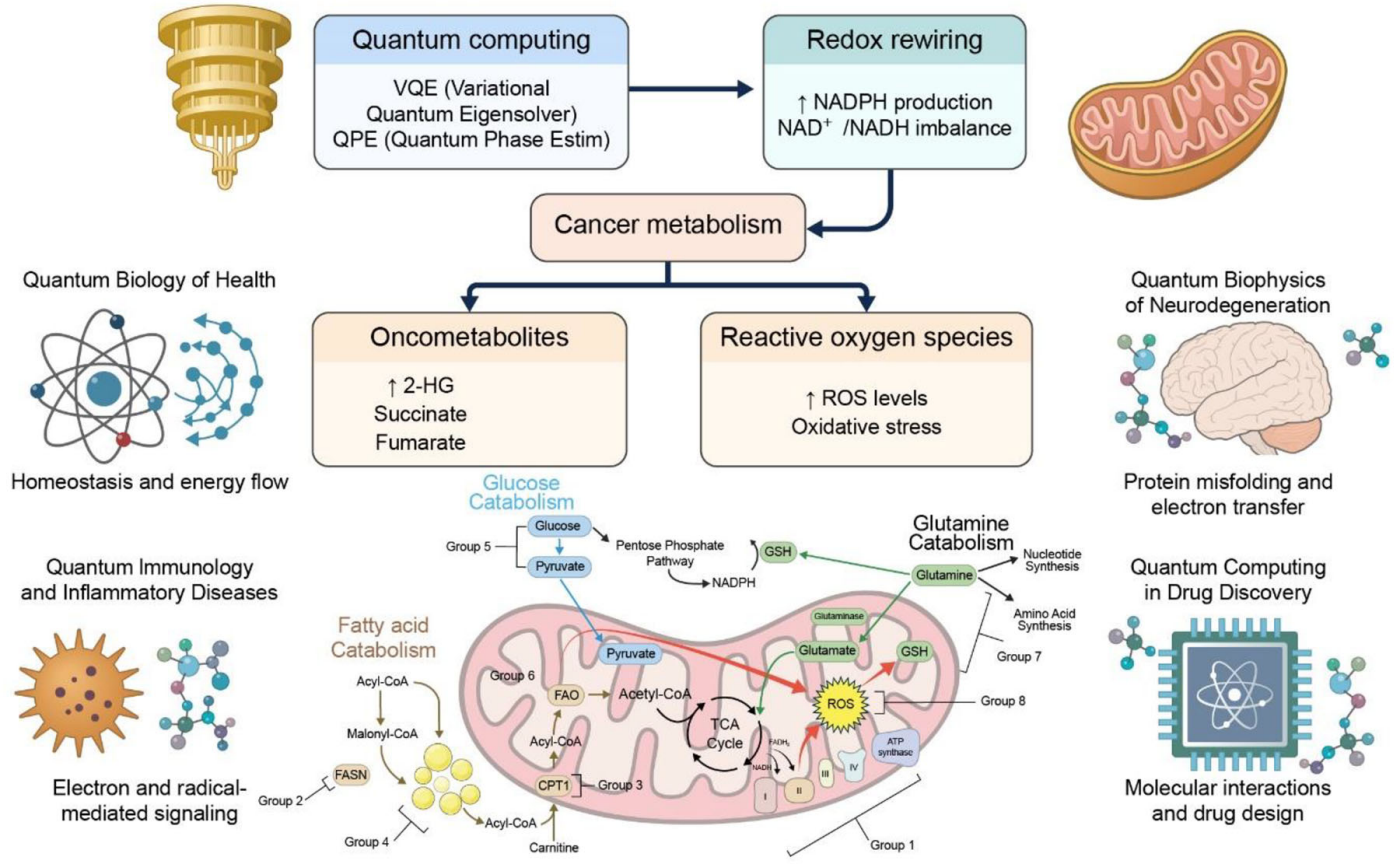
Quantum‐informed model of cancer metabolism and redox rewiring. This schematic illustrates how quantum computing and redox biology converge to reshape cancer metabolism. At the top, quantum algorithms such as the variational quantum eigensolver (VQE) and quantum phase estimation (QPE) enable ab initio modelling of redox enzymes and metabolic cofactors, providing quantum‐level insights into NAD^+^/NADH imbalance and increased NADPH production (redox rewiring). These alterations in redox homeostasis drive oncogenic metabolic reprogramming, characterized by elevated oncometabolites (2‐hydroxyglutarate, succinate and fumarate) and increased reactive oxygen species (ROS) generation, leading to oxidative stress. The mitochondrial diagram depicts the interplay between glucose, fatty acid and glutamine catabolism, showing how electron flow through the tricarboxylic acid (TCA) cycle and oxidative phosphorylation influences ROS formation and NADPH balance. Peripheral panels highlight broader biomedical implications of quantum biology: (left) homeostatic energy flow and radical‐mediated signalling in health and immune regulation; (right) quantum biophysics of neurodegeneration, including protein misfolding and electron transfer and quantum computing–driven drug discovery through molecular interaction modelling. Together, these concepts underscore how quantum computation can mechanistically decode cancer metabolism by linking electronic‐level processes with systems‐level bioenergetics.

The QPE algorithm provides a more precise calculation of molecular energy eigenvalues, especially in small biomolecules. In a biomedical context, QPE is valuable for benchmarking redox potentials (Δ*E*) in enzymatic systems and for mapping the energetic consequences of conformational mutations in cancer‐associated proteins such as p53 and KRAS. These benchmarks are difficult to obtain through classical DFT alone, particularly in systems with multireference character.^7^, ^60^ In the realm of enzyme kinetics and metabolism, PCET plays a central mechanistic role. Quantum simulations of PCET can uncover tunnelling pathways and vibronic coupling mechanisms in metabolic enzymes like lactate dehydrogenase or succinate dehydrogenase, both of which are implicated in oncogenic metabolic rewiring.^61^, ^62^, ^63^, ^64^, ^65^ Such models support mechanistic interpretation of data from metabolic flux analysis (MFA) and redox biosensor assays.^47^ These links are visually summarized in Figure 2, which maps VQE, QPE and PCET to concrete biomedical domains‐including mitochondrial dysfunction, redox enzymology and cancer metabolism‐thereby making the abstract quantum algorithms more approachable to life science audiences. As illustrated in Figure 2, the core quantum algorithms‐VQE, QPE and PCET‐based simulations‐are mapped directly onto specific biomedical targets, including mitochondrial redox enzymes, cancer‐associated metabolic pathways and protein–ligand interaction networks. This schematic demonstrates how abstract quantum formalisms translate into experimentally measurable biological observables such as redox potentials, tunnelling rates and binding free energies, thereby providing a mechanistic bridge between quantum computation and molecular medicine.

In summary, quantum computing provides qualitatively new capabilities that transcend the limitations of classical methods, enabling the direct simulation of quantum phenomena such as tunnelling, electron correlation and spin dynamics. These principles lay the foundation for applying quantum technologies to complex biological systems. In the next section, we translate this theoretical understanding into practice by exploring how quantum computing is being applied across a range of biomedical domains—from cancer metabolism and protein folding to health and disease.^66^, ^67^, ^68^, ^69^, ^70^

Before introducing the QEC framework, it is important to situate this work within the rapidly growing body of quantum biomedical research. Over the past decade, multiple independent lines of investigation have demonstrated that quantum technologies can provide biologically meaningful and experimentally testable insights into living systems.

In the domain of QML, hybrid quantum classifiers and variational quantum neural networks have been applied to bulk and single‐cell transcriptomic datasets, achieving cancer subtype classification and biomarker discovery performance that in several studies equals or exceeds that of state‐of‐the‐art classical models. These results indicate that quantum‐enhanced feature spaces can capture non‐linear and high‐order relationships in omics data that are difficult to access with classical methods alone.

In parallel, quantum chemical simulations have been used to model redox‐active biological cofactors, including iron–sulfur clusters, flavins and metalloproteins. Using algorithms such as the VQE and QPE, recent studies have predicted redox potentials and electronic coupling parameters within approximately  .1 eV of experimental values‐surpassing the accuracy of standard DFT for strongly correlated systems. These advances are directly relevant to mitochondrial respiration, cancer metabolism and oxidative stress biology. A third line of progress has emerged from quantum sensing, particularly nitrogen–vacancy (NV) centre‐based probes that can detect magnetic fields, redox potentials and reactive oxygen species (ROS) in living cells with nanoscale resolution. These technologies enable direct experimental measurement of quantum‐relevant observables, such as spin states and redox fluctuations, thereby providing a physical bridge between quantum simulations and biological reality. Finally, quantum protein modelling and quantum‐informed docking approaches have begun to map conformational energy landscapes and ligand–receptor interactions with improved fidelity over classical force‐field methods, offering new strategies for drug discovery and mutation effect prediction.

The QEC framework proposed in this review does not replace these prior achievements but integrates them into a coherent translational architecture. Quantum algorithms generate first‐principles electronic and spin‐level observables, experimental platforms validate and parameterize these predictions, and clinical datasets provide the phenotypic and therapeutic context. In this way, the QEC pipeline synthesizes existing successes across quantum computation, quantum sensing and biomedical experimentation into a unified workflow capable of supporting mechanistic, testable and clinically relevant quantum medicine (Table 1).

**TABLE 1:** Comparative summary of representative quantum–biomedical studies.

Study/Application	Dataset/Biological system	Quantum method	Achieved performance/key findings	Classical benchmark/comparison
Cancer subtype classification^71^	TCGA bulk RNA‐seq (breast, lung, colon)	Hybrid quantum SVM/QNN	>90% accuracy in classifying cancer subtypes with reduced feature dimensionality	Classical SVM: ∼85% accuracy with higher feature count
Biomarker discovery^72^	CTLA4 pathway gene expression profiles	Variational quantum classifier	Identified novel biomarker sets correlating with immunotherapy response	Conventional ML missed low‐abundance features
NV‐centre sensing of redox state^73^	In vitro mitochondrial samples; live‐cell models	NV‐centre quantum sensor	Detects Δ*E* shifts <10 mV and ROS flux in real time with nanoscale resolution	Electrochemical sensors: ≥50 mV detection threshold, lower spatial resolution
Redox potential calculation in Fe–S clusters)^42^, ^44^, ^74^	Bioinorganic model clusters (6–12 atoms)	VQE/QPE	Predicted reduction potentials within .1 eV of experimental values	DFT: typical error ∼.2–.3 eV
Protein–ligand interaction modelling^75^	EGFR kinase variants	Quantum computational chemistry (UCC–VQE)	Binding affinity predictions matched experimental Δ*G* within .3 kcal/mol	Classical docking ∼1.0 kcal/mol deviation
Neoantigen–MHC binding^76^	MHC‐II peptide complexes	Semi‐empirical QM/quantum‐informed modelling	Improved binding affinity prediction accuracy by ∼20% over classical scoring functions	Standard MHC prediction tools showed lower sensitivity
Quantum spin biology and ROS control^77^	ROS production in cell culture under magnetic fields	Quantum spin dynamics simulation	Revealed triplet‐singlet transitions affecting ROS yield	Classical kinetics failed to reproduce spin‐dependent effects

*Note*: This table provides a structured synthesis of key experimental and computational studies at the intersection of quantum technologies and biomedicine. Each entry summarizes the biological dataset or system investigated, the quantum methodology applied, the major performance outcomes achieved and corresponding classical benchmarks for comparison (Table 1). Together, these examples illustrate how quantum computing, quantum sensing and quantum machine learning have been applied across multiple biomedical domains—including cancer metabolism, protein–ligand interactions, redox sensing and spin‐dependent ROS regulation. The comparative perspective highlights both the current achievements and the remaining challenges, providing readers with a clear view of the field's progress and potential translational impact. The reported performance values reflect the validated accuracy ranges and resolution limits provided in the original publications, rather than full cohort‐level statistical summaries, which are not yet systematically available in the current quantum–biomedical literature.

Abbreviations: DFT, density functional theory; NV, nitrogen–vacancy; QPE, quantum phase estimation; ROS, reactive oxygen species; VQE, variational quantum eigensolver.

## 3 APPLICATIONS OF QUANTUM COMPUTING IN MOLECULAR BIOMEDICINE

### 3.1 Quantum biology of health‐homeostasis and energy flow

Health is sustained through quantum‐regulated processes that preserve molecular order and energetic balance. Electron tunnelling, PCET and spin‐selective radical reactions enable enzymes and cellular networks to coordinate energy conversion and biochemical signalling with remarkable precision.

Quantum algorithms, such as the VQE and QPE, allow first‐principles modelling of these fundamental processes, reproducing experimental redox potentials within <.1 eV accuracy and predicting electron coupling strengths beyond classical DFT. These approaches also help elucidate how redox fluctuations contribute to metabolic regulation and oncogenic transformation, providing new insight into how cells maintain‐or lose‐energetic homeostasis. We view health not merely as a biochemical steady state but as a quantum‐stabilized equilibrium sustained by coherence, tunnelling and dynamic resonance across molecular networks. Biology, in this view, is a living expression of quantum order.^78^, ^79^, ^80^, ^81^, ^82^

### 3.2 Quantum metabolism and redox rewiring in cancer

Cancer metabolism represents one of the most fertile frontiers for the application of quantum biomedicine. Malignant transformation is not merely a genomic or biochemical event—it reflects a reorganization of quantum‐regulated redox and energy‐transfer networks within the cell. ET, PCET,^83^ and spin‐selective radical reactions^84^ govern how metabolic enzymes maintain NAD^+^/NADH balance, ATP synthesis and ROS homeostasis.^85^ When these quantum processes become dysregulated, they manifest as hallmark metabolic phenotypes of cancer‐including aerobic glycolysis (Warburg effect), glutamine addiction and the accumulation of oncometabolites such as 2‐hydroxyglutarate (2‐HG), succinate and fumarate.^86^, ^87^, ^88^, ^89^, ^90^, ^91^, ^92^, ^93^

Quantum algorithms‐including the VQE^94^, ^95^ and QPE‐enable ab initio simulation of redox enzymes and cofactor clusters that underpin these pathways. Likewise, quantum simulations of PCET in dehydrogenases (e.g., IDH1/2, SDH and FH) elucidate tunnelling barriers and vibronic coupling mechanisms that explain how specific oncogenic mutations alter catalytic kinetics and favour accumulation of oncometabolites. From a systems perspective, this ‘redox rewiring’ constitutes a quantum phase transition in metabolism‐where coherent electron flow through respiratory chains gives way to decohered, stochastic redox cycles driving tumourigenesis. The NAD^+^/NADH and NADP^+^/NADPH ratios, central to biosynthetic and antioxidant control, are thus emergent quantum observables reflecting collective electronic states of metabolic enzymes. Quantum sensing with NV centres in diamond now allows in situ detection of these redox shifts at millivolt precision,^96^ offering experimental validation of simulated quantum parameters.^36^, ^97^, ^98^, ^99^, ^100^

Quantum models further illuminate the interplay between metabolic ROS and signalling. Radical pair mechanisms‐spin‐correlated electron pairs within oxidoreductases‐mediate singlet‐triplet transitions that modulate ROS yield. Such spin‐dependent kinetics explain why cancer cells exhibit elevated yet tunable ROS levels, balancing oxidative stress with proliferative signalling. Simulations of these radical dynamics on NISQ devices reveal that subtle magnetic‐field perturbations can shift ROS partitioning, hinting at magnetosensitive vulnerabilities exploitable for therapy.^101^, ^102^, ^103^ Integrating quantum computation, sensing and machine learning establishes a comprehensive framework for ‘quantum oncology’. VQE‐derived redox observables can feed QML classifiers trained on metabolomic and transcriptomic data to stratify tumours by redox phenotype or predict response to NADPH‐targeted drugs. This synergy bridges the quantum scale of electron tunnelling with the cellular scale of metabolic reprogramming.^104^, ^105^, ^106^

Ultimately, cancer metabolism exemplifies how disease emerges from decoherence of life's quantum order. Restoring coherent redox coupling‐through pharmacologic modulation of electron flow or quantum‐guided enzyme design‐may define the next generation of metabolic therapeutics. In this view, quantum computing does not merely simulate cancer; it deciphers its energetic code.^107^, ^108^, ^109^, ^110^

### 3.3 Quantum biophysics of neurodegeneration

**FIGURE 3:**
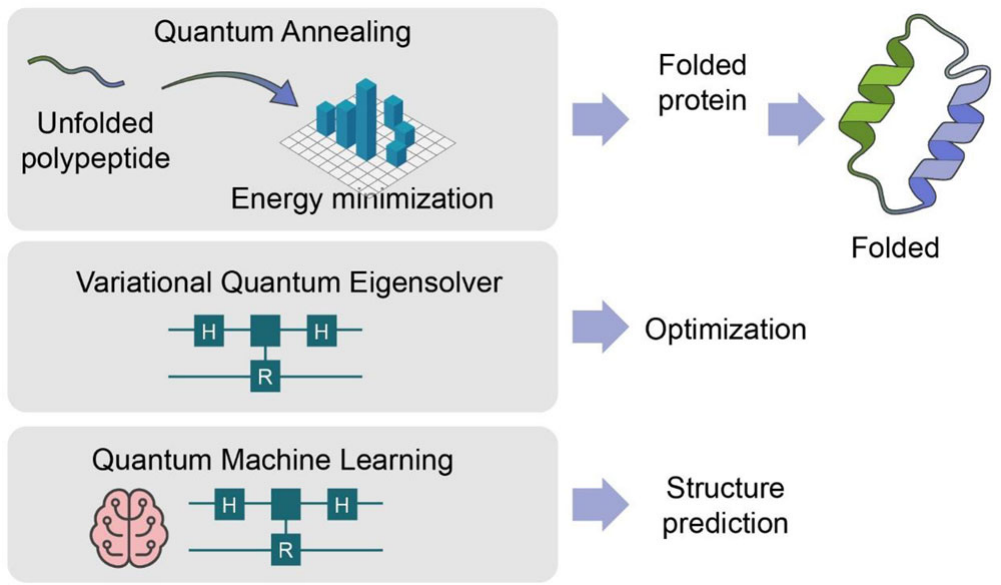
Quantum computational workflow for protein folding and misfolding analysis. This figure illustrates the integration of three major quantum computational paradigms—quantum annealing (QA), variational quantum eigensolver (VQE) and quantum machine learning (QML)—for modelling protein folding, misfolding and conformational transitions. Quantum annealing minimizes the potential energy landscape to identify low‐energy conformers, whereas VQE refines local minima and computes electronic structures underlying hydrogen bonding and π–π interactions. QML models then learn complex folding patterns from quantum‐derived observables, enabling prediction of disease‐associated misfolding pathways. Together, these approaches establish a unified framework for simulating protein energetics and dynamics at quantum resolution, providing mechanistic insights into neurodegenerative diseases such as Alzheimer's and Parkinson's.

Neurodegenerative diseases represent a class of disorders deeply linked to electron and spin dynamics in proteins.^111^ Misfolding of amyloidogenic proteins (α‐synuclein, tau, amyloid‐β) alters their electronic structure and aggregation pathways.^112^ Quantum mechanical simulations of hydrogen‐bond networks and π–π interactions reveal energy landscapes responsible for folding errors and oligomer formation.^50^ By integrating VQE‐based energy mapping and QITE, researchers can compute conformational energy minima and transition barriers that define protein stability.^113^ Quantum simulations of ET in mitochondrial complexes also help explain neuronal vulnerability to oxidative stress, as seen in Parkinson's and Alzheimer's disease. These insights bridge molecular electronic perturbations with clinical symptoms, forming a quantum‐level understanding of neurodegeneration‐where loss of coherence and redox imbalance manifest as cellular dysfunction and cognitive decline. We propose that neurodegeneration represents not only a biological failure but a quantum decoherence event‐a breakdown of coherence and energy flow that echoes across molecular and cognitive scales (Figure 3). Figure 3 situates quantum‐level phenomena‐such as electron tunnelling, spin dynamics and coherence‐within the context of protein misfolding and neurodegenerative disease. By linking alterations in electronic structure to conformational instability and aggregation pathways, this figure supports the central argument that molecular‐scale quantum effects can propagate upward to cellular dysfunction and clinical pathology.

Beyond neurodegeneration, quantum coherence may underlie aspects of neural synchronization and cognitive integration. Emerging studies in quantum neurobiology suggest that coherent spin and redox oscillations in microtubules or mitochondrial networks could contribute to perception, memory formation and consciousness. Integrating these models within biomedical frameworks could open a new frontier‐quantum cognition as a physiological process rather than a philosophical abstraction.

### 3.4 Quantum immunology and inflammatory diseases

Immunity depends on electron‐and radical‐mediated signalling. Enzymes, like nitric oxide synthase and myeloperoxidase, use quantum tunnelling and spin transitions to generate ROS and reactive nitrogen species,^114^ which regulate immune activation and pathogen defence. Quantum computing now allows mechanistic modelling of these enzyme systems, elucidating how spin coupling or redox imbalance triggers inflammation or autoimmunity.^115^ For instance, quantum models can simulate cytokine–receptor binding energetics, offering insight into molecular recognition in rheumatoid arthritis, lupus and chronic inflammatory diseases. Through quantum simulations and quantum sensing, it becomes possible to visualize immune signalling at the electronic level, providing tools for developing targeted immunomodulatory drugs.^116^

Immune regulation can be described in quantum–mechanical terms, involving coordinated interactions of spin, charge and electromagnetic fields that link physical processes to physiological immune function. Quantum immunology invites a new way of understanding immune balance‐not as a static biochemical state but as a dynamic quantum order maintained through coherent spin and redox interactions.^117^ Disruption of this order may underlie chronic inflammation and autoimmunity. Future research integrating quantum sensing^118^ and simulation could visualize immune dynamics at the electronic level, linking molecular spin states with macroscopic immune responses. Ultimately, restoring coherence and redox symmetry may become a new therapeutic principle, positioning immune regulation as an emergent expression of quantum resilience in living systems.^119^

### 3.5 Quantum perspectives on infectious diseases

Pathogens exploit quantum processes to survive oxidative stress, metabolize nutrients and evade host defences. Quantum simulations of microbial redox enzymes (e.g., cytochromes, flavoproteins) reveal how bacteria regulate electron flow to maintain metabolic flexibility under antibiotic pressure. In viral systems, quantum‐informed structural modelling helps predict RNA‐protein interactions and spike conformational switching, informing antiviral drug design. By integrating VQE, QML and redox dynamics, researchers can explore how mutations affect enzyme catalysis or drug binding‐offering predictive insight into emerging antibiotic resistance and viral mutation fitness.

A summary of experimental strategies for validating quantum‐derived observables in various biomedical contexts is provided in Table 2.

**TABLE 2:** Experimental validation strategies for quantum biomedical models.

Quantum modelling target	Biomedical context	Quantum observable	Validation technique	Clinical relevance
Electron transfer kinetics in mutated metabolic enzymes^120^, ^121^, ^122^, ^123^, ^124^	Cancer metabolism (e.g., IDH1, SDH and FH mutations)	k_ET, redox potential (Δ*E*)	Metabolic flux analysis (MFA), NAD^+^/NADH quantification	Identifying redox‐sensitive drug targets in tumours
Redox potential shifts in Fe–S clusters (Complex I/II)^125^, ^126^	Mitochondrial dysfunction, Leigh syndrome, Parkinson's	Δ*E*, spin‐coupling patterns	Cryo‐EM, EPR spectroscopy, NV‐centre redox imaging with sham (no‐probe) and FCCP‐collapsed membrane potential controls	Stratifying mitochondrial disease subtypes
Conformational stability in p53 and other IDPs^127^, ^128^, ^129^	Protein misfolding in cancer, neurodegeneration	Energy landscapes, decoherence signatures	Structural biology (NMR, cryo‐EM), aggregation assays	Predicting misfolding‐induced oncogenic transitions
PCET in oxidoreductases (e.g., lactate dehydrogenase)^130^, ^131^, ^132^	Cancer glycolysis, immune cell energetics	Tunnelling barrier, vibronic coupling	pH/redox‐sensitive biosensors, isotope labelling studies	Optimizing metabolic enzyme inhibitors
Quantum spin states in radical pairs^133^, ^134^	ROS signalling, magnetic sensitivity in cancer	Spin correlation, triplet‐singlet transitions	Time‐resolved fluorescence, magnetogenetics	Elucidating non‐classical redox signalling mechanisms
Drug‐binding affinity in mutant kinases^135^, ^136^	Targeted therapy (e.g., EGFR, KRAS and BRAF variants)	Ligand–residue interaction energy (Δ*G*_bind)	Quantum‐informed docking + biochemical binding assays	Predicting drug resistance and refining targeted therapies
Neoantigen–MHC binding affinity^137^, ^138^, ^139^, ^140^	Cancer immunotherapy	Electronic structure of peptide–MHC complexes	Immunopeptidomics, quantum MHC binding simulation	Enhancing neoantigen prediction for personalized vaccines

*Note*: This table summarizes how quantum computational predictions can be experimentally validated across diverse biomedical contexts. Each row links a specific quantum modelling target—such as electron transfer kinetics, redox potential shifts, conformational stability, proton‐coupled electron transfer (PCET) or spin‐state dynamics—to its corresponding biological context, measurable quantum observables and standard validation techniques. Together, these examples demonstrate how quantum‐derived predictions (e.g., redox potential Δ*E*, tunnelling barriers, spin transitions or ligand‐binding free energies) can be tested through established experimental approaches, including metabolic flux analysis, cryo‐EM, EPR spectroscopy, biosensor assays and magnetogenetics. This integrative framework bridges theoretical quantum modelling with translational biomedical applications, enabling the identification of redox‐sensitive drug targets, the prediction of oncogenic protein misfolding and the development of personalized therapeutic strategies and experimental validation strategies for quantum biomedical models.

Abbreviations: cryo‐EM, cryo‐electron microscopy; ROS, reactive oxygen species.

We see infection not only as a biochemical battle but as a quantum competition‐where coherence, tunnelling and entanglement become evolutionary tools for survival. Furthermore, quantum‐informed modelling can accelerate vaccine design and mutation prediction by simulating how electronic‐level changes in viral antigens alter binding affinities with host receptors or antibodies.^141^ By coupling quantum chemistry with machine learning, researchers can forecast potential escape mutations and identify energetically stable antigenic configurations before they emerge in circulation.^142^, ^143^

### 3.6 Quantum simulation of redox enzymes and free radicals

Beyond protein folding and mitochondrial energy transfer, a growing body of evidence suggests that quantum effects underlie the catalytic function of redox enzymes and the behaviour of biological free radicals. In particular, enzyme‐catalysed ET reactions often rely on PCET^62^, ^131^, ^144^ and quantum tunnelling,^144^, ^145^ especially in oxidoreductases such as dehydrogenases, peroxidases and DNA repair enzymes.^131^ These quantum phenomena help explain reaction rates that exceed classical expectations and are fundamental to understanding the redox balance in cancer metabolism. Additionally, spin biology,^146^ a subfield examining quantum spin dynamics in biological systems, has gained attention for its relevance to ROS generation^147^ and magnetosensitivity in cells. Radical pair mechanisms,^109^, ^110^ which involve spin‐correlated electron pairs, are thought to influence biochemical outcomes depending on magnetic field interactions.^148^ These mechanisms may contribute to differential redox states in cancer cells or to ROS‐mediated DNA damage and signalling pathways.^149^ Quantum computing provides a promising framework to model these complex spin‐dependent interactions and tunnelling processes with high accuracy and scalability. By simulating enzyme–substrate complexes at the electronic level or by reconstructing spin Hamiltonians in radical pair systems, quantum algorithms could reveal new therapeutic insights‐particularly in diseases such as cancer, where redox regulation and free radical signalling are disrupted.^149^, ^150^

In our view, redox enzymes act as nature's quantum processors‐where spin dynamics, tunnelling and coherence shape the flow of biological information and energy.

### 3.7 Quantum sensing for biomedical diagnostics

Although quantum computing offers computational breakthroughs, quantum sensing provides new ways to measure biological phenomena at unprecedented resolution. Quantum sensors,^96^, ^118^ especially those based on NV centres in diamond,^98^, ^151^ can detect minute changes in magnetic fields, electric fields, temperature and redox states—all of which are central to disease biology.^96^ In cancer, for instance, tumour microenvironments are characterized by steep gradients in oxygen concentration, pH, redox potential and ion fluxes. Conventional sensors frequently fall short in resolving the fine spatial or temporal details needed to detect such microheterogeneities. Quantum sensors,^152^ by contrast, can operate at the nanoscale, potentially allowing the real‐time mapping of mitochondrial redox dynamics, metabolic fluxes or ROS generation in live cells.^153^ In neurodegenerative diseases such as Parkinson's and Alzheimer's, where mitochondrial dysfunction and iron dysregulation are prominent, quantum sensors could be employed to: Track metal ion concentrations (e.g., Fe^2+^, Cu^2+^) within neurons or glial cells, monitor free radical dynamics that contribute to oxidative damage and detect early redox imbalance before structural degeneration occurs. Furthermore, quantum sensors may assist in drug discovery, providing a direct readout of molecular interactions, target engagement or local biochemical changes in response to therapeutic compounds.^99^, ^154^, ^155^, ^156^, ^157^, ^158^, ^159^ Their ability to non‐invasively probe single molecules or organelles opens new frontiers in diagnostics, personalized medicine and mechanistic pharmacology.^160^ Ultimately, quantum sensing complements quantum computing by generating high‐fidelity data that can feed into quantum simulations, completing the loop from measurement to modelling.^96^, ^161^, ^162^, ^163^, ^164^ We envision diagnostics evolving from passive observation to quantum participation—where measurement itself becomes an active part of the biological process it observes.

### 3.8 Quantum machine learning in biomedical data science

The explosion of biomedical data‐from single‐cell transcriptomes to proteomic landscapes and clinical imaging‐demands computational frameworks that can extract patterns beyond classical limits. QML, which integrates quantum computing with statistical learning, offers a powerful new approach for identifying disease mechanisms, stratifying patients and guiding therapeutic decisions.^71^, ^165^, ^166^ Unlike classical machine learning, QML can exploit the exponential expressivity of quantum states and perform feature transformations in high‐dimensional Hilbert spaces. This is particularly valuable in biomedical domains where relationships between features are non‐linear, high‐order and entangled across multiple modalities.^167^, ^168^ Promising applications include cancer subtype classification using QML‐enhanced dimensionality reduction on bulk and single‐cell RNA‐seq datasets, prediction of immunotherapy response via hybrid quantum classifiers trained on multi‐omic tumour profiles and detection of metastasis‐prone phenotypes by identifying latent molecular patterns from imaging‐genomics data. Moreover, QML can be combined with quantum chemistry and simulation results, enabling closed‐loop workflows where: Quantum‐derived observables (e.g., Δ*E*, *k*_ET, spin states) feed into QML models as input features, QML identifies biologically relevant quantum subsystems or cofactors most predictive of disease, and the resulting insights guide the design of follow‐up quantum simulations or sensing experiments. Early implementations of QML have shown promise in areas such as protein structure classification, compound screening and gene signature refinement. As more quantum hardware becomes available and specialized quantum kernels are developed, QML could evolve into a central tool for precision medicine‐offering interpretable models that align with the mechanistic depth of quantum simulations.^167^, ^168^, ^169^, ^170^ QML is expected to function not only as a computational tool but also as a cognitive extension of biomedicine, enabling machines to infer biological patterns within quantum–mechanical representations.

### 3.9 Quantum computing in drug discovery

Quantum computing is set to redefine the landscape of drug discovery by enabling the simulation of intricate molecular interactions with a level of precision and scale far beyond the reach of classical approaches. Conventional computational methods often struggle to capture the full complexity of large biomolecules, which hampers the speed and accuracy of therapeutic development. In contrast, quantum systems‐leveraging foundational principles such as superposition and entanglement‐can efficiently probe vast molecular conformational spaces, opening new avenues for identifying and optimizing drug candidates through iterative refinement.^171^, ^172^ Simulating MD is a fundamental use case for quantum computing in this context. These simulations allow for detailed insights into how drugs interact with biological targets at the quantum level, thus informing the design of more selective and potent therapies. Promising progress has already been made in applying this approach to oncology, where quantum models are beginning to aid in the discovery of next‐generation cancer treatments.^173^ In addition, the integration of quantum computing with machine learning is shaping a transformative new paradigm. This integration enables the construction of highly predictive models of molecular behaviour and accelerates the identification of novel therapeutic compounds.^174^ The synergy between quantum algorithms and data‐driven methods is expected to drastically shorten the timeline for drug discovery and broaden the scope of treatable diseases.^171^, ^175^, ^176^ Another key advantage lies in enhancing hit validation‐the process of confirming promising lead compounds. Quantum simulations offer more reliable predictions of molecular activity, helping to reduce false positives and streamline early stage drug development. This is especially crucial for complex diseases like cancer, where nuanced molecular insights are essential for therapeutic success. Although current NISQ devices face technical constraints such as decoherence and limited qubit capacity, continuous advancements in quantum hardware and algorithm development are rapidly addressing these challenges. Recent breakthroughs have demonstrated quantum simulations of biologically relevant molecules with impressive accuracy, signalling a transformative potential in the drug discovery pipeline. As the technology matures, quantum computing is poised to become a cornerstone of next‐generation therapeutics, enabling the creation of more effective, personalized treatments across a spectrum of diseases.^114^, ^177^, ^178^, ^179^, ^180^, ^181^, ^182^ Integrating quantum simulation outputs with pharmacogenomic data may allow personalized prediction of drug efficacy and toxicity. Quantum algorithms could simulate drug metabolism in variant‐specific enzyme isoforms, bridging precision medicine with quantum pharmacology.

The examples presented in this section demonstrate that quantum computing is not merely a theoretical tool‐it is already beginning to transform how we understand, model and manipulate biological systems. From uncovering metabolic vulnerabilities to predicting protein misfolding pathways and decoding genomic variation, quantum approaches offer unprecedented resolution into disease mechanisms. However, translating these computational advances into clinical impact requires bridging the gap between in silico modelling and experimental validation. The next section discusses strategies to integrate quantum simulations with experimental pipelines and clinical applications, ensuring that theoretical predictions are grounded in biological reality.^179^, ^183^, ^184^ Recent advances in quantum simulation suggest new avenues for integrating molecular modelling and chemical synthesis in drug discovery. In this context, quantum biomedicine is increasingly discussed as a potential paradigm for shifting medicine from a largely descriptive discipline toward a computationally grounded understanding of biological systems.

## 4 BRIDGING QUANTUM SIMULATIONS WITH EXPERIMENTAL AND CLINICAL APPLICATIONS

Although quantum computing offers unparalleled capabilities for modelling biological complexity, its full potential will only be realized when these computational insights are linked to experimental validation and clinical translation. This section focuses on the critical bridge between theory and practice. We discuss how hybrid computational pipelines can integrate quantum simulations with classical methods, how quantum predictions can guide laboratory assays and organoid models, and how experimental data can refine quantum models to improve accuracy and relevance.

**FIGURE 4:**
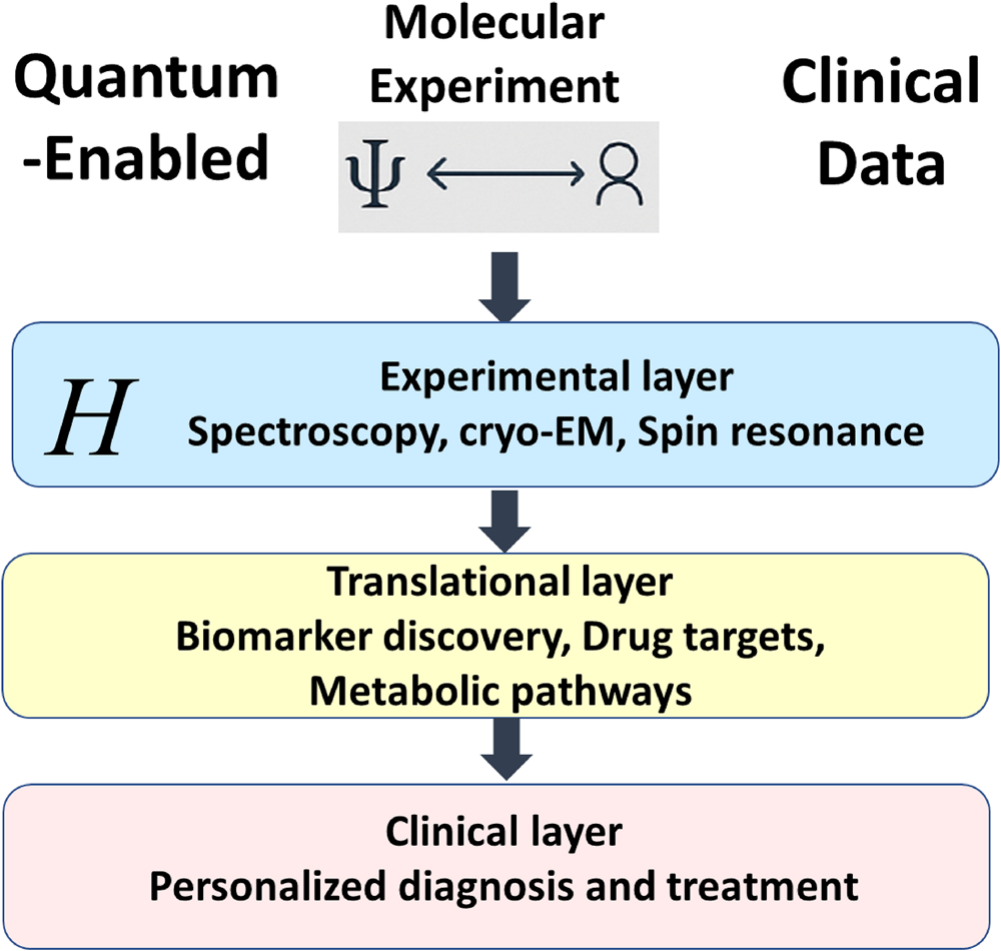
Translational framework linking quantum simulations, molecular experiments and clinical applications. This schematic illustrates the multi‐layered quantum‐experimental–clinical (QEC) pipeline envisioned in quantum biomedicine. At the top, quantum‐enabled molecular experiments integrate quantum computation and biophysical measurements to model molecular Hamiltonians (H) that describe fundamental processes such as electron transfer, proton‐coupled electron transfer (PCET) and spin dynamics. These theoretical predictions are validated in the experimental layer through spectroscopy, cryo‐electron microscopy (cryo‐EM) and spin resonance techniques, ensuring that computed quantum observables (e.g., redox potentials, tunnelling rates, spin states) correspond to measurable biological quantities. The results inform the translational layer, where insights are applied to biomarker discovery, drug target identification and metabolic pathway analysis—bridging computational models with biological function. Finally, the clinical layer translates these validated models into personalized diagnostic and therapeutic strategies, integrating patient‐derived omics and clinical data. Together, these layers form an iterative feedback loop that connects quantum simulation, experimental validation and clinical translation‐laying the foundation for a quantum‐enabled biomedical ecosystem.

Rather than replacing classical approaches, quantum computing serves as a valuable complement to them. When used together, they can enable simulations that are both precise and computationally feasible. As summarized in Figure 4, the translational workflow connects quantum simulations with experimental validation and clinical application‐from in silico prediction of redox‐sensitive vulnerabilities to metabolic flux assays and patient‐derived organoid models.^9^, ^185^, ^186^

By combining both approaches, a hybrid strategy enables researchers to leverage the advantages of each: DFT^187^, ^188^ can serve as an efficient pre‐screening tool for identifying relevant molecular geometries or cofactor configurations. Quantum algorithms, such as the VQE^189^ or QPE, can then be applied to biologically significant targets derived from omics analyses or structural biology. Insights from quantum simulations can be integrated into broader computational workflows, including kinetic models of metabolic flux, AI‐driven platforms for virtual drug screening and protein–ligand docking frameworks for drug–target interaction studies. In addition, QML offers a novel bridge between complex biological datasets and quantum observables. By extracting hidden patterns‐such as high‐entropy cellular states or cofactor‐binding signatures‐QML can help predict and interpret quantum‐relevant behaviours within biological systems.

To enhance the translational relevance of quantum biomedicine, we provide specific case studies that demonstrate how quantum simulations can predict experimentally verifiable outcomes. For instance, modelling IDH1/2 mutations in gliomas enables quantum computation of oncometabolite redox shifts (e.g., 2‐hydroxyglutarate accumulation), which can be validated via MFA and mass spectrometry. Similarly, simulations of p53 misfolding under mutational stress predict local electron density rearrangements and hydrogen‐bond disruption, which are amenable to cryo‐electron microscopy (cryo‐EM) or NMR validation. These pathways are illustrated in Figure 4, which outlines a translational pipeline linking quantum observables to experimental and clinical endpoints.^1^, ^171^, ^190^, ^191^

### 4.1 Opportunities with NISQ devices

Although today's NISQ devices remain limited by qubit coherence, error rates and circuit depth, they already provide a valuable testbed for biomedical applications. NISQ hardware enables proof‐of‐concept simulations of redox reactions, electron tunnelling and spin‐dependent biochemical processes that are computationally intractable on classical systems. For instance, hybrid VQE–DFT frameworks can approximate reaction energetics in enzyme active sites, whereas QITE and quantum annealing approaches can explore conformational landscapes of biomolecules and predict energetically feasible states relevant to folding diseases. Furthermore, NISQ platforms can be coupled with quantum sensors and lab‐on‐chip biophysical assays, creating an experimental–computational feedback loop. This enables direct benchmarking of quantum models against empirical observables such as redox potential shifts, radical pair kinetics or conformational transitions under physiological conditions. In this way, NISQ‐based quantum simulations serve as translational bridges that connect theoretical quantum chemistry with measurable biomedical phenomena.^192^, ^193^, ^194^, ^195^

### 4.2 Envisioning a quantum‐enabled biomedical ecosystem

Realizing quantum biomedicine requires transformation at the ecosystem level. This shift must occur across multiple dimensions‐cultural, educational, institutional and technological. Culturally, it demands genuine transdisciplinary collaboration, where physicists learn the language of biology and clinicians develop an intuitive understanding of quantum concepts such as Hamiltonians. Educationally, integrated curricula in quantum life sciences, along with dedicated postdoctoral fellowships and MD–PhD pathways in quantum biology, are essential to train the next generation of researchers. Establishing interdisciplinary training programmes‐spanning quantum information science, molecular biology and clinical medicine‐will be essential. Graduate curricula in quantum biomedicine should integrate computational modules with laboratory practicums, preparing researchers to navigate both qubit design and biological complexity.

Institutionally, establishing centres for quantum health science, consortia for quantum disease modelling and shared quantum infrastructures will provide the foundation for sustained progress. Technologically, the field will benefit from open‐source quantum libraries and biologically annotated Hamiltonian databases that bridge computation with experimental biology, ensuring that quantum biomedicine evolves as a cohesive and collaborative scientific enterprise.

Bridging quantum computation with biological experimentation is essential for transforming abstract predictions into clinically actionable insights. By creating iterative feedback loops between simulation and validation, we can accelerate the development of quantum‐informed diagnostics, therapeutics and personalized medicine strategies. With this translational foundation established, the final section turns toward future perspectives‐examining the remaining challenges, key research questions and the long‐term vision for quantum‐enabled biomedicine. To transition from simulation to real‐world utility, quantum biomedicine must evolve into a multi‐layered research ecosystem that couples quantum computation with molecular experiments and clinical data.

At the experimental layer, quantum‐derived Hamiltonians can be parameterized with spectroscopic, electrochemical and cryo‐EM data, ensuring that model predictions align with physical observables. Techniques, such as ultrafast spectroscopy, spin resonance and single‐molecule redox imaging, can provide critical benchmarks for validating quantum models of biological processes. At the translational layer, these validated models can inform biomarker discovery, drug target identification and metabolic pathway analysis by integrating with multi‐omics datasets and patient‐derived cell models.^196^

Finally, at the clinical layer, hybrid quantum–classical algorithms and QML can be deployed for personalized diagnosis and treatment optimization, identifying patient‐specific redox or spin signatures associated with disease progression or therapeutic response. This integrated QEC pipeline creates a feedback system in which theoretical predictions guide experiments, experimental data refine simulations, and both collectively inform clinical decision‐making. Such an iterative approach accelerates the translation of quantum principles from molecular simulations to precision medicine.

### 4.3 Toward a quantum‐enabled biomedical ecosystem

Realizing this vision requires infrastructure and policy innovation. Establishing quantum–biomedical research hubs equipped with shared quantum computing access, biophysical instrumentation and clinical databases will foster collaboration across traditionally siloed disciplines. Standardization efforts‐such as biologically annotated Hamiltonian repositories and open‐source simulation toolkits‐will be crucial for reproducibility and cross‐validation across laboratories. Equally important is the development of ethical and interpretative frameworks for clinical quantum applications, ensuring that algorithmic outputs are both biologically meaningful and clinically actionable.

Together, these initiatives will transform quantum biomedicine from a theoretical discipline into a translational science that directly informs experimental biology and clinical medicine.

### 4.4 Governance, ethics and sustainable implementation

The emergence of quantum biomedicine demands not only technological innovation but also a new framework of governance, ethics and sustainability. As quantum computing begins to influence diagnostics, drug discovery and clinical decision‐making, questions of data integrity, algorithmic interpretability and patient privacy will become central.^197^ Establishing clear ethical standards for how quantum‐derived predictions are validated, communicated and implemented in medicine is essential for building public trust and clinical reliability. Quantum–biomedical algorithms should be transparent, reproducible and auditable, ensuring that outputs remain biologically meaningful and clinically actionable. Beyond ethics, sustainable implementation must be considered. Quantum computing infrastructures are inherently resource‐intensive‐requiring specialized cryogenic environments, significant power consumption and complex maintenance. To ensure environmental and economic sustainability, global research networks should prioritize shared infrastructures, cloud‐based quantum access and green computing strategies. Open‐access quantum platforms can democratize participation, allowing laboratories without in‐house quantum hardware to engage in quantum–biomedical simulations, fostering a globally inclusive scientific community. Equally important is governance at the institutional and policy level. The establishment of national and international frameworks for quantum–biomedical research will be critical to harmonize standards, regulate clinical translation and facilitate data interoperability. Dedicated ethics boards should oversee the development and deployment of quantum diagnostic or therapeutic tools, providing continuous evaluation of risk–benefit balance. Policies promoting transparency, accountability and equitable access will prevent the emergence of technological monopolies and ensure that quantum medicine benefits humanity as a whole. Finally, inclusivity and education must guide this transformation. Building a global quantum–biomedical commons‐comprising open datasets, interoperable models and shared best practices‐will nurture collaboration and accelerate discovery. Integrating ethical training and sustainability awareness into quantum–biomedical curricula will prepare future scientists to navigate the social and moral dimensions of this emerging discipline. In this way, governance and ethics become not constraints but catalysts‐anchoring quantum biomedicine as a socially responsible, environmentally conscious and globally accessible science that serves both knowledge and humanity.

## 5 FUTURE PERSPECTIVES AND CHALLENGES

Despite its transformative potential, quantum biomedicine remains an emerging and evolving field with important assumptions and limitations that must be explicitly acknowledged. First, current quantum hardware operates in the NISQ regime, which imposes constraints on qubit number, coherence time and circuit depth. As a result, most biomedical applications of quantum computing rely on hybrid quantum–classical workflows rather than fully fault‐tolerant quantum simulations.

Second, the accuracy of quantum simulations in biology depends critically on the construction and parameterization of biologically relevant Hamiltonians. Mapping complex cellular environments‐including solvent effects, protein dynamics and multiscale interactions‐onto tractable quantum models remains a major challenge and often requires simplifying assumptions. Consequently, quantum‐derived observables, such as redox potentials, ET rates or spin states, must be interpreted in conjunction with experimental validation rather than as standalone predictions.

Third, quantum algorithms, such as VQE, QPE and QML models, face scalability and convergence limitations. Although proof‐of‐concept studies demonstrate impressive accuracy for small molecular systems and reduced‐dimensional biological models, extending these approaches to full proteins, organelles or whole‐cell systems will require substantial advances in hardware, algorithms and error mitigation.

Fourth, translational barriers remain significant. Integrating quantum outputs into clinical workflows requires standardized data formats, reproducible pipelines and robust experimental benchmarks. Moreover, the cost, accessibility and technical expertise required for quantum computing may initially limit its deployment to specialized research centres.

Finally, ethical, regulatory and governance considerations must accompany technical progress. The interpretability of hybrid quantum–AI models, the reliability of probabilistic outputs, and equitable access to quantum‐enabled healthcare technologies are critical issues that must be addressed before clinical adoption.

By explicitly articulating these assumptions and limitations, this review provides a realistic foundation for evaluating both the promise and the current boundaries of quantum biomedicine and for guiding its responsible and evidence‐based development toward clinical translation.

Despite these promising advances, the field of quantum biomedicine remains in its infancy. Significant challenges‐including hardware scalability, algorithmic efficiency and biological data integration‐must be addressed before quantum tools can become routine components of biomedical research. Recognizing these limitations is essential not only for realistic expectations but also for guiding collaborative efforts that will transform today's conceptual promise into tomorrow's clinical reality.

As outlined throughout this work, quantum computing holds transformative potential across a wide spectrum of biomedical domains. Quantum modelling illuminates disrupted redox dynamics and identifies metabolic vulnerabilities in cancer, whereas quantum‐powered simulations of protein folding reveal subtle electronic shifts that underlie misfolding and allosteric regulation. By integrating classical and quantum computational workflows, systems biology models can now be refined with mechanistic depth informed directly by quantum observables. In the study of redox enzymes and radical signalling, quantum biology fills a long‐standing theoretical gap by explaining spin‐dependent, non‐equilibrium biochemical processes. Moreover, advances in quantum sensing bring us closer to detecting biomarkers, metabolites and even protein conformations with unprecedented sensitivity and spatial resolution. Finally, QML enables the analysis of complex, high‐dimensional omics and clinical datasets in ways that uncover hidden disease subtypes and novel therapeutic targets. Together, these developments mark the convergence of physics and medicine, defining a truly quantum‐informed framework for understanding life and disease.

Together, these pillars constitute a quantum‐native perspective on biomedicine‐one that acknowledges the inadequacy of purely classical frameworks to capture life's full complexity. Biology is not an approximation of physics; it is a realization of quantum principles, structured into molecules, networks and ultimately health and disease.^40^, ^96^ But realizing this vision will require more than hardware improvements. It demands a restructuring of our scientific culture, where biophysicists, chemists, clinicians and quantum theorists co‐design experiments and algorithms, guided by biological questions. It calls for educational reform to train the next generation of quantum–biomedical thinkers who can bridge disciplines and domains. The advancement of quantum biomedicine demands proactive institutional investment through specialized research hubs, strategic funding pathways and the development of interoperable platforms for modelling and validating quantum‐level biological phenomena.^198^, ^199^ This is not a time to hesitate‐it is a time to lead with conviction. The integration of quantum mechanics into biomedicine will not unfold passively; it must be actively forged by those who understand that the next revolution in medicine transcends molecules‐it embraces quantum principles. We call on the biomedical community to step forward with urgency and vision. The potential gains are extraordinary: breakthrough therapies, deeper mechanistic insights and a radically new paradigm for understanding the foundations of life.

In conclusion, quantum biomedicine is rapidly evolving from conceptual speculation into a transformative scientific discipline. Its ability to capture fundamental quantum phenomena in biological systems‐from electron tunnelling and spin dynamics to redox regulation and conformational transitions‐2holds enormous promise for understanding disease mechanisms and developing novel therapies. Achieving this vision will require sustained interdisciplinary collaboration, advances in hardware and algorithms, integration with omics and clinical data, and new frameworks for experimental validation. With continued investment and collective effort, quantum technologies are poised to become foundational tools in precision medicine, enabling a deeper and more complete understanding of life at the quantum level.^6^, ^177^, ^200^, ^201^, ^202^, ^203^

### 5.1 A decadal roadmap for quantum biomedicine: from theory to clinical translation

**FIGURE 5:**
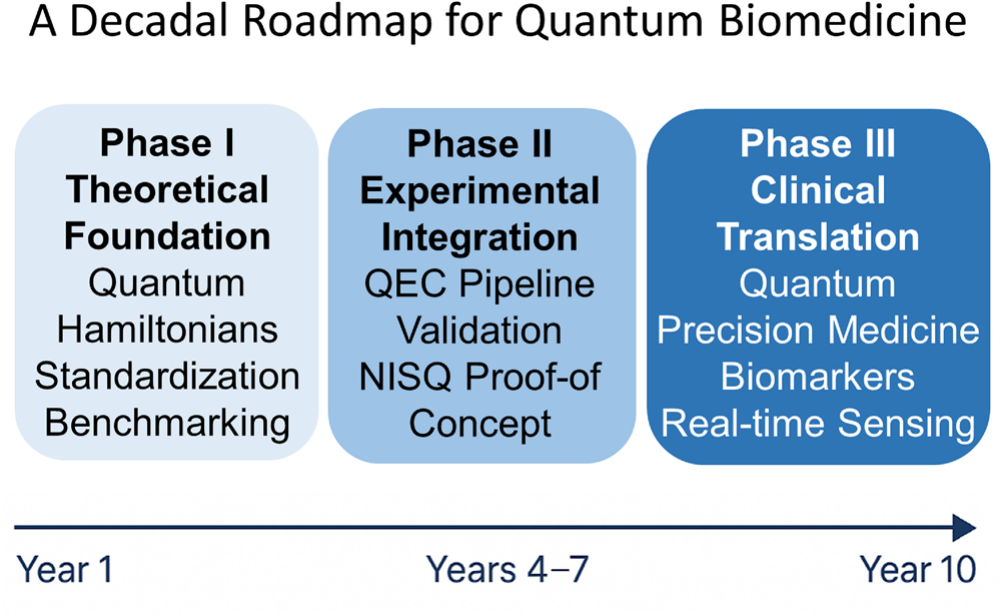
A decadal roadmap for quantum biomedicine: from theory to clinical translation. This figure outlines a 10‐year roadmap for the evolution of quantum biomedicine, progressing from theoretical formulation to clinical application. Phase I (Years 1–3) focuses on developing biologically relevant Hamiltonians, model standardization and open quantum repositories. Phase II (Years 4–7) integrates quantum simulations with experimental validation, establishing the quantum–experimental–clinical (QEC) feedback loop. Phase III (Years 8–10) envisions the clinical translation of quantum principles through quantum biomarkers, quantum‐enhanced diagnostics and personalized quantum‐informed therapies. Together, these phases define a coherent strategy to transform quantum biomedicine from conceptual theory into translational precision medicine.

Realizing the promise of quantum biomedicine will not happen spontaneously—it requires a deliberate, structured roadmap that connects abstract quantum theory to measurable biological and clinical realities (Figure 5). We envision this transformation unfolding over the next decade through three progressive phases: building a theoretical foundation, achieving experimental validation and advancing clinical translation.

#### 5.1.1 Phase I (Years 1–3): theoretical foundation and model standardization

In the first phase, the field must establish a rigorous theoretical backbone that defines what it truly means to ‘compute biology’ in quantum terms. Researchers should focus on developing biologically relevant Hamiltonians that explicitly incorporate redox coupling, spin–orbit interactions and conformational free‐energy landscapes. These models will bridge the gap between the mathematics of quantum mechanics and the physical chemistry of living systems. Equally essential is the creation of computational benchmarks that connect quantum observables—such as tunnelling rates or spin coherence times—to measurable biological quantities, including reaction kinetics and redox potentials. This will provide the quantitative scaffolding necessary to test theory against experiment. To ensure reproducibility and cross‐laboratory collaboration, the establishment of standardized open‐access repositories for biomolecular quantum data—covering Hamiltonians, coupling constants and potential energy surfaces—is indispensable. Through these coordinated efforts, quantum biomedicine can consolidate its language, unify its mathematical structure and evolve from scattered theoretical insights into a coherent scientific discipline.

#### 5.1.2 Phase II (Years 4–7): experimental integration and quantum–biological validation

Once the theoretical framework is in place, the next challenge is to merge quantum computation with empirical biophysics. During this phase, NISQ devices will serve as experimental testbeds for simulating small biological modules such as flavoproteins and Fe–S clusters. The resulting outputs can be directly compared with spectroscopic and electrochemical measurements, allowing theoretical predictions to be confronted with experimental truth. Beyond computation, quantum algorithms must be integrated with high‐resolution experimental tools‐including ultrafast spectroscopy, EPR and cryo‐EM‐to validate quantum models of electron tunnelling and redox‐driven conformational change. Most importantly, we advocate for the construction of QEC pipelines, in which quantum simulations inform molecular experiments, and those experimental results, in turn, refine the simulations. Integrating these with omics and systems biology data will establish a dynamic feedback loop between theory and observation. In our view, this phase represents the moment when quantum biomedicine transitions from abstraction to embodiment‐when ideas begin to touch biological matter.

#### 5.1.3 Phase III (Years 8–10): clinical translation and quantum‐informed precision medicine

The final phase will bring quantum biomedicine to the frontlines of human health. Here, QML will play a central role in integrating high‐dimensional omics and clinical datasets to uncover hidden disease subtypes and predict therapeutic responses. We anticipate the identification of quantum biomarkers‐molecular signatures defined by spin, redox or coherence properties‐that can distinguish health from disease with unprecedented sensitivity. A clinically grounded example of QML is provided by a hospital‐based cohort study in which a hybrid quantum support vector machine was applied to predict mortality risk in early onset colorectal cancer patients using electronic medical records from 2008 to 2020 (*N* = 1253). The cohort included 93 clinical variables spanning age, sex, tumour stage, treatment history and laboratory measurements, and the quantum‐enhanced model achieved an AUROC of approximately  .90, demonstrating the feasibility of deploying quantum biomarkers on real patient data.^204^ In parallel, hybrid quantum–classical classifiers have also been trained on large public patient cohorts such as TCGA (e.g., glioma and breast cancer), where quantum variational circuits and quantum kernels successfully stratified tumour subtypes and identified clinically relevant biomarkers including IDH1, PTEN and EGFR.^205^

At the technological frontier, quantum‐enabled diagnostic platforms combining sensing, computation and AI could enable real‐time clinical decision‐making based on quantum‐level biological dynamics. These systems will bridge molecular behaviour and medical outcomes, embodying the ultimate goal of translational quantum medicine. Finally, to sustain such progress, institutional and cultural infrastructure must evolve through the establishment of dedicated quantum health centres, interdisciplinary training programmes and supportive policy frameworks. We believe that by the end of this decade, these collective efforts will transform quantum biomedicine from a conceptual frontier into a functional pillar of precision medicine, capable of illuminating the quantum architecture of life itself.

We do not see quantum biomedicine as a distant abstraction but as the natural next stage in humanity's quest to understand life. Over the next 10 years, the field must move from simulating molecules to decoding the very logic of living matter. This is not merely technological progress—it is a philosophical shift toward seeing biology as quantum reality expressed in form and function.

### 5.2 Ethical and societal frontiers of quantum medicine

As quantum technologies converge with human biology, they bring not only computational power but also profound ethical and philosophical implications. Quantum simulations capable of representing molecular, neural or even organismal processes challenge long‐standing definitions of life, consciousness and individuality. When human physiology can be modelled with quantum precision, questions arise about data sovereignty, the moral status of simulated entities and the boundaries between biological and digital existence. These developments call for a re‐examination of what it means to ‘treat’, ‘enhance’, or even ‘replicate’ life in a quantum era.

Equitable access represents another urgent concern. Quantum resources remain concentrated within a few industrial and academic centres, risking the emergence of a new ‘quantum divide’ in global healthcare. To prevent inequities between quantum‐enabled and classical medical systems, international cooperation must ensure fair allocation of quantum infrastructure, cloud access and educational opportunities. Building a globally distributed network of quantum–biomedical hubs can democratize innovation and foster collective progress. Beyond science, quantum biomedicine will redefine global healthcare economics by shifting innovation from molecule‐centred industries to information‐centred ecosystems. Ensuring equitable access to quantum infrastructure will determine whether this revolution narrows or widens the health gap across nations.^197^

Furthermore, interpretability and accountability will be crucial as hybrid quantum–classical algorithms begin informing clinical decisions. Physicians and patients alike must understand the probabilistic and non‐deterministic nature of quantum outputs. Regulatory frameworks should therefore evolve to encompass algorithmic transparency, model explainability and continuous post‐deployment auditing‐ensuring that quantum predictions remain both scientifically valid and ethically defensible. As quantum–AI integration accelerates, new ethical dimensions emerge around quantum data ownership, model autonomy and algorithmic accountability. Future quantum medical AI systems must ensure human oversight, preventing epistemic opacity where decisions arise from unexplainable quantum processes.

Finally, quantum medicine invites humanity to reconsider its relationship with nature at the most fundamental level. By revealing the coherence, entanglement and resonance that underlie living systems, it blurs the distinction between physics and physiology, computation and consciousness. Navigating these frontiers responsibly will require a new synthesis of science, ethics and philosophy‐one that views quantum medicine not merely as a technological revolution, but as a reflection of humanity's deeper quest to understand and sustain life itself.

## AUTHOR CONTRIBUTIONS

Ji‐Yong Sung wrote the original draft. Ji‐Yong Sung and Jae‐Ho Cheong designed and wrote the paper. Both authors contributed to the interpretation of the results. Both authors have read and agreed to the published version of the manuscript.

## CONFLICT OF INTEREST STATEMENT

The authors declare no conflicts of interest.

## FUNDING INFORMATION

Korean ARPA‐H Project through the Korea Health Industry Development Institute (KHIDI), Ministry of Health & Welfare, Republic of Korea (grant number: RS‐2025‐25456722), This research was supported by the “Regional Innovation System & Education (RISE)” through the Seoul RISE Center, funded by the Ministry of Education (MOE) and the Seoul Metropolitan Government. (2025‐RISE‐01‐022‐05).

## ETHICS STATEMENT

The authors have nothing to report.

## CONSENT

The authors have nothing to report.

## AI USE DECLARATION

AI tools were used only for English grammar correction and language polishing.

## Data Availability

The authors have nothing to report.
